# Effect of Various Surface Treatments on the Push-Out Bond Strength of Fiber Posts to Root Dentin

**DOI:** 10.7759/cureus.51323

**Published:** 2023-12-30

**Authors:** Janani Karunakaran, Deepa N Thangaraj, Sebeena Mathew, Karthick Kumaravadivel, Boopathi Thangavel

**Affiliations:** 1 Conservative Dentistry and Endodontics, Chettinad Dental College and Research Institute, Chennai, IND; 2 Conservative Dentistry and Endodontics, KSR Institute of Dental Science and Research, Tiruchengode, IND

**Keywords:** titanium tetrafluoride, silanization, sandblasting, surface treatment, fiber posts

## Abstract

Background

This study aimed to evaluate the effect of various surface treatments on the push-out bond strength of fiber posts to root dentin.

Methodology

A total of 96 single-rooted premolars were decoronated and obturated and post spaces were prepared for 9 mm. The canals were irrigated with 17% ethylenediaminetetraacetic acid followed by distilled water. The samples were divided into the two following groups based on the type of fiber posts used: Group I - glass fiber post (Reforpost size 1) and Group II: quartz fiber post (Quartzix Added Posts number 1). Further, each group was divided into four subgroups based on the surface treatments (A: no treatment (control); B: silanization; C: 4% titanium tetrafluoride (four minutes) followed by silanization; D: sandblasting followed by silanization). After surface treatments, posts were cemented using self-adhesive resin cement (RelyX U200). Three 2 mm thick slices were obtained and push-out tests were done. Failure modes were analyzed under a stereomicroscope. The surface morphology of the posts was analyzed with a scanning electron microscope. Data were analyzed using a one-way analysis of variance.

Results

Treating the posts with airborne particle abrasion (sandblasting) followed by silanization showed the highest bond strength. The coronal level of the root showed the highest bond strength compared to the middle and apical levels. Adhesive failures between the resin cement and dentin were found to be the highest.

Conclusions

Sandblasting followed by silanization produced the highest bond strength. The coronal level of the root showed the highest bond strength. Adhesive failures were the highest followed by mixed failures.

## Introduction

Endodontic treatment of teeth removes much of the coronal tooth structure, which affects the structural durability and functionality of the tooth. They generally require a core for the restoration of tooth function. For the retention of the core, an intraradicular post is required [1]. Custom cast posts have been traditionally used for this purpose, but they have been avoided due to several factors such as the requirement for two appointments, non-conservative preparation, need for temporization, chances of corrosion, lack of adhesion, and, most importantly, the lack of esthetics [2]. Many studies have reported that the physical properties of cast metal posts and cores weaken the roots of teeth instead of reinforcing them [3]. Thus, as an alternative to cast metal posts and cores, various nonmetallic posts have emerged.

Since the invention of carbon fiber posts, they have gained popularity for their biocompatibility, ease of placement, improved corrosion resistance, esthetics, and easy removal for retreatments [4]. The elastic moduli of fiber posts approximate those of dentin, and restoring teeth with these materials reduces the risk of root fracture [5]. Fiber-reinforced composite (FRC) posts possess a continuous network of strengthening fibers in a resin matrix. Matrix polymers between these fibers are generally epoxy resins and are highly cross-linked [6]. This makes the fiber posts less reactive and creates difficulty in bonding these posts to tooth structure [7]. However, these posts can be adhesively bonded to the root canal using resin cements, leading to the formation of a dentin-post adhesive system, known as tertiary monoblock [8].

Although they possess various advantages, a major drawback of fiber-reinforced posts is that they cannot achieve close adaptation, unlike the custom-made posts in canals with varied morphology [9]. The second most common cause of failure of these posts is their debonding. The most common adhesive bond failure occurs at the resin-dentin interface [10].

Various surface treatments have been studied such as (1) treatments that tend to produce a chemical bonding (coating with primers), (2) treatments that tend to produce micromechanical bonding (sandblasting and etching), and (3) a combination of micromechanical and chemical bonding to provide better retention of fiber posts to root dentin [11]. These surface treatments tend to increase the surface area for bonding by exposing the glass fibers [12]. Thus, this study aims to enhance the retention of fiber posts to root dentin.

Titanium tetrafluoride (TiF_4_) seems to have varied applications in dentistry-fissure sealant, desensitizer, ceramic etchant, and varnish to prevent enamel demineralization [13].

Of the various surface treatments, silanization is a widely used and simple procedure, which can be done before cementation of the post, but its effect on the fiber posts’ bond strength is inconclusive in that some studies report that silanization improves the bonding between the fiber posts and resin cement [14], whereas some report that silanization is insufficient to produce a better bond [15]. Thus, we aimed to study chemical and micromechanical treatment with silanization.

This study was designed to analyze the impact of various surface treatments on the push-out bond strength of fiber posts cemented with self-adhesive resin cement to the root dentin. The null hypothesis was that no significant differences would exist between the push-out bond strength of various surface treatments of fiber posts.

## Materials and methods

**Figure 1:**
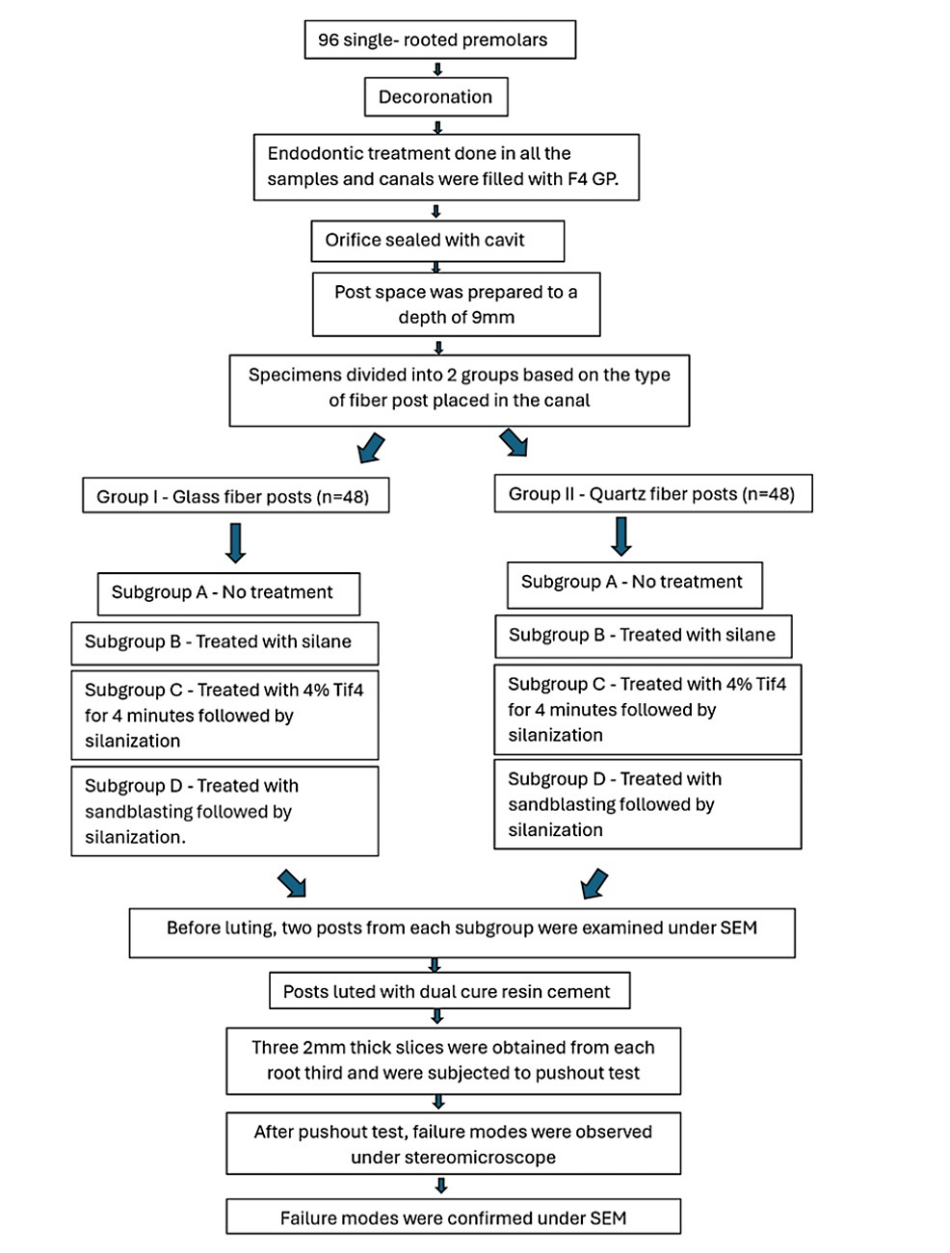
Flowchart of the study methodology.

Ethical clearance for this study was obtained from the Institutional Ethical Committee, KSR Institute of Dental Science and Research (approval number: 206/KSRIDSR/EC/2018). The methodology flowchart is presented in Figure 1. A total of 96 single-rooted premolars with similar anatomy and fully formed apices were selected and stored in 0.9% saline solution. The root surface was cleaned using an ultrasonic scaler. All teeth were decoronated to a standard length of 14 mm using a diamond disc under water coolant.

Access opening was done in each sample, and the working length was calculated using an initial apical file. Biomechanical preparation was done with Protaper Gold Rotary files (Dentsply Maillefer, Switzerland) up to F4. During preparation, the canals were irrigated with 2 mL of 5.25% NaOCl. Final irrigation was done with 5 mL of 17% ethylenediaminetetraacetic acid (EDTA) for one minute, and the final rinse was done with 5 mL of distilled water.

The root canals were dried with paper points (Dentsply Maillefer, Switzerland) and then filled with gutta-percha (Dentsply Maillefer, Switzerland) and AH-Plus sealer (AH Plus; Dentsply, Konstanz, Germany) using a single-cone obturation technique. The orifice was sealed with Cavit (3M ESPE, Germany). The samples were stored at 37°C at 100% relative humidity for a week.

The post space preparation was done to a depth of 9 mm with #3 Peeso reamer (Mani Inc, Japan) leaving apical 4-5 mm of gutta-percha. After post space preparation, the canals were irrigated with 17% EDTA, followed by distilled water, and dried with paper points.

**Figure 2:**
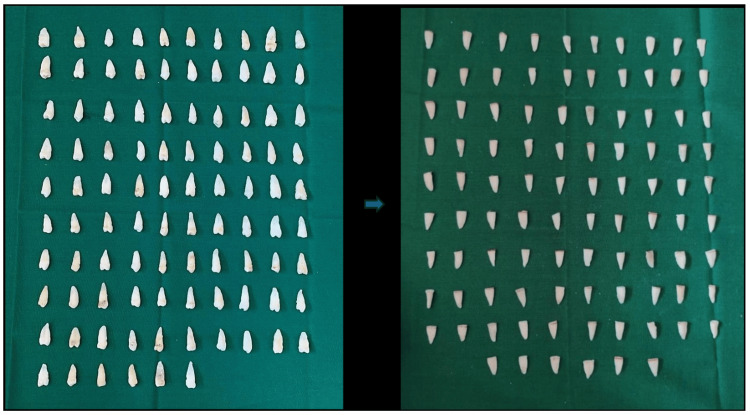
Ninety-six samples and samples after decoronation.

**Figure 3:**
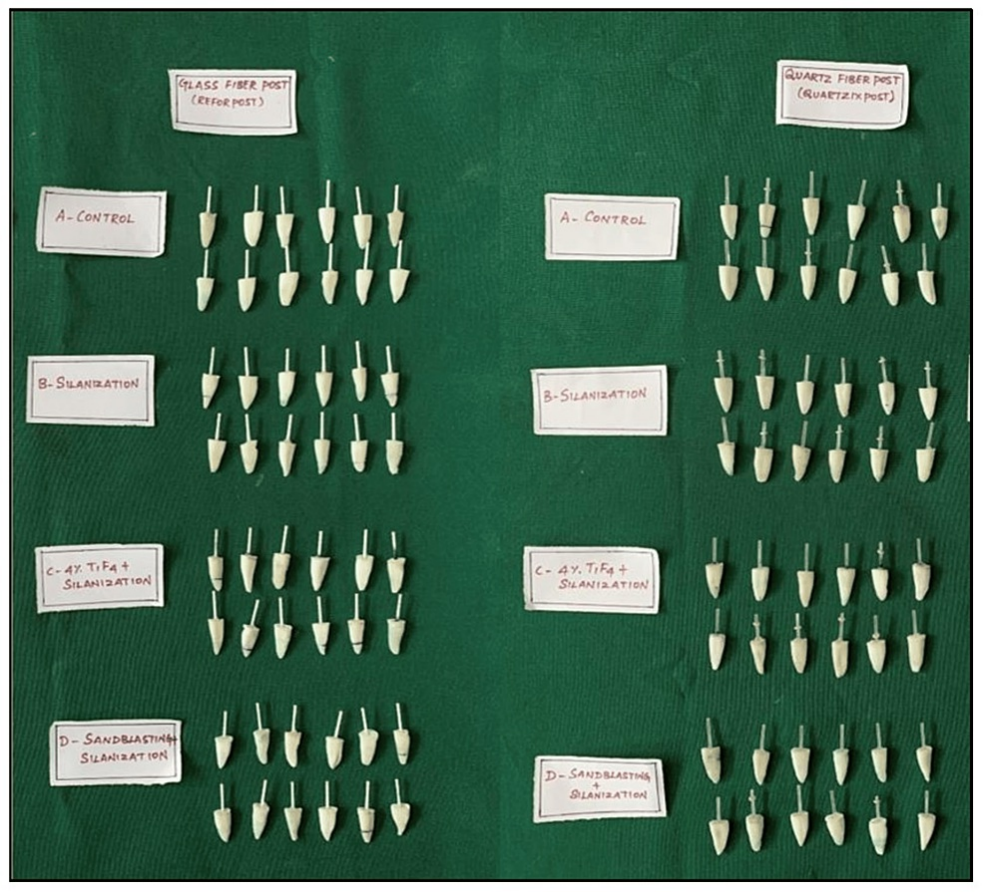
Samples divided into subgroups after surface treatment.

Then, samples (Figure 2) were randomly divided into two main groups of 48 specimens each based on the type of fiber posts placed in the canal. Each group was further divided into four subgroups (12 specimens in each) (Figure 3) based on the surface treatment performed. The randomization was done by an expert who was completely unaware of the study.

**Figure 4:**
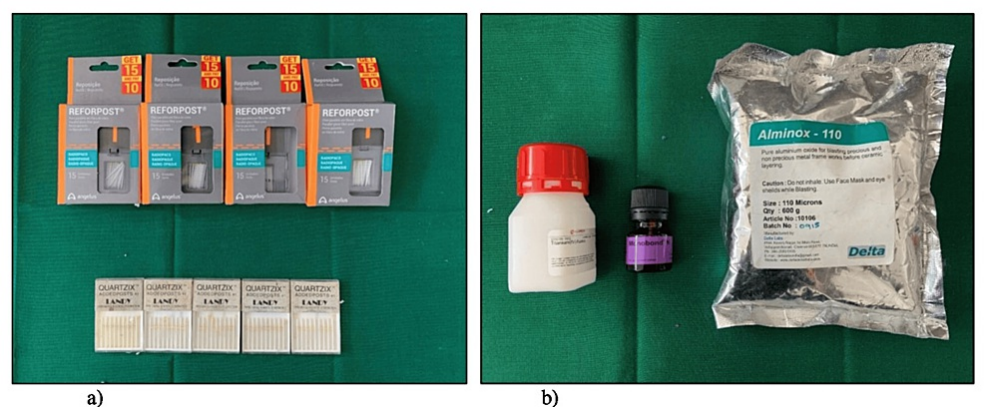
(a) Glass fiber posts and quartz fiber posts. (b) Surface treating agents.

Group I included glass fiber posts (Reforpost of size 1 (Angelus, Londrina, Brazil)), and Group II included quartz fiber posts (Quartzix Added Posts; number 1 (Landy, Swiss Dental Products of Distinction)). The groups were divided into the following subgroups: subgroup A - no treatment, subgroup B - treated with a silane coupling agent, subgroup C - treated with 4% TiF_4_ for four minutes followed by silanization, and subgroup D - sandblasting followed by silanization (Figures 4a, 4b).

In groups IA and IIA, the posts were not surface treated. In groups IB and IIB, the silane coupling agent (Monobond - N, Ivoclar - Vivadent, Schaan, Liechtenstein) was applied with a micro brush and air dried at room temperature for 60 seconds. In groups IC and IIC, the posts were immersed in 4% TiF_4_ for four minutes before being silanated for 60 seconds. TiF_4_ powder (Aldrich Chemical Company, WI, USA) was dissolved in distilled water to determine the required concentration. In groups ID and IID, the posts were air abraded using 110 aluminum oxide particles for five seconds from 1 cm. After sandblasting, the posts were rinsed with running water and cleaned ultrasonically in distilled water for five minutes.

All posts were luted with dual cure resin cement (Relyx U200; 3M ESPE) and light cured for 40 seconds with an LED curing light. The specimens were kept in distilled water storage for 24 hours at 37°C and then evaluated for push-out test.

Three 2 mm slices of each root third were obtained using a diamond disc under water cooling. Push-out test was done using a universal testing machine with load applied in an apical-coronal direction at a cross-head speed of 0.5 mm/minute until bond failure occurred. The push-out pin was placed at the center of the post, and various sizes of pins were used to correspond to the diameter of the post at different root thirds. The highest force that caused the expulsion of the post was noted as the bond failure point and was recorded in Newtons (N).

Bond strength (MPa) = Load value recorded in N/Area of the bonded interface.

Bonded surface area = 2πrh, where π is constant, r is the radius of the perforated area, and h is the height of the specimen in mm.

After the push-out test, samples were examined under a stereomicroscope to identify the type of failure. Failures were divided into adhesive (between cement and post or cement and dentin), cohesive (on cement or post), or mixed [4].

Before luting the posts after surface treatment, two surface-treated posts from each group (one a glass fiber post and the other a quartz fiber post) were examined under a scanning electron microscope (SEM) (Carl Zeiss, USA). The specimens were gold sputter coated and vacuum packed in argon for two minutes and at 25 mA to obtain a 100 A uniform stratum of golden powder. The failure modes were confirmed under SEM.

Statistical analysis

Results were analyzed using SPSS version 17.0 (SPSS Inc., Chicago, IL, USA). Descriptive statistics were performed, and continuous values were presented as mean and standard deviation. Mean was compared by one-way analysis of variance (ANOVA) followed by a post-hoc test performed using a Bonferroni test. Statistical significance was set at the 5% level.

## Results

Table 1 and Table 2 show the statistically significant difference (p < 0.05) between the groups at the coronal level and middle level, respectively.

**Table 1:** Comparison of mean push-out bond strength (megapascals) between groups at the coronal level. One-way analysis of variance shows a statistically significant difference (p < 0.05) between the groups at the coronal level.

Coronal	Mean	Standard deviation	Standard error	Minimum	Maximum	Pvalue
Group IA	4.01	1.49	0.43	2.13	7.41	0.001*
Group IB	4.46	1.27	0.37	1.27	6.10	
Group IC	4.30	1.37	0.39	1.95	5.99	
Group ID	5.42	1.79	0.52	3.00	8.82	
Group IIA	3.54	1.49	0.43	0.97	6.30	
Group IIB	4.47	2.19	0.63	2.38	8.95	
Group IIC	4.65	1.74	0.50	2.56	8.69	
Group IID	6.42	1.47	0.41	3.88	8.45	

**Table 2:** Comparison of mean push-out bond strength (megapascals) between groups at the middle level. One-way analysis of variance shows a statistically significant difference (p < 0.05) between the groups at the middle level.

Middle	Mean	Standard deviation	Standard error	Minimum	Maximum	P-value
Group IA	2.99	1.10	0.32	0.83	4.20	0.008*
Group IB	4.09	1.54	0.44	1.13	6.97	
Group IC	4.31	1.54	0.44	0.86	6.64	
Group ID	5.17	2.49	0.72	1.72	9.53	
Group IIA	3.22	2.47	0.71	0.77	9.39	
Group IIB	3.37	1.90	0.55	0.71	7.39	
Group IIC	3.18	1.45	0.42	1.58	6.65	
Group IID	5.21	1.58	0.44	3.23	7.88	

Group IID showed the highest bond strength, followed by group ID, IIB, IB, IIC, and IC. Sandblasting with silanization showed the highest bond strength with both the glass and quartz fiber posts followed by silane-treated and TiF_4_-treated quartz fiber posts. Quartz fiber posts produced higher bond strength than glass fiber posts.

Table 3 displays the percentage of different failure modes of all groups after push-out tests at the coronal, middle, and apical levels. There was a predominance of adhesive failures in coronal and middle levels but not in the apical level, where mixed failures were found to be higher in some groups. Cohesive failures were found to be the least at all levels. The coronal end of the root showed the highest values in comparison with middle and apical levels.

**Table 3:** Percentage of different failure modes after push-out tests.

		Group IA	Group IB	Group IC	Group ID	Group IIA	Group IIB	Group IIC	Group IID
Coronal	Adhesive	7	8	7	8	11	9	8	11
		58.30%	66.70%	58.30%	66.70%	91.70%	75.00%	66.70%	84.60%
	Cohesive	1	0	0	1	0	1	0	0
		8.30%	0.00%	0.00%	8.30%	0.00%	8.30%	0.00%	0.00%
	Mixed	4	4	5	3	1	2	4	2
		33.30%	33.30%	41.70%	25.00%	8.30%	16.70%	33.30%	15.40%
Middle	Adhesive	6	4	5	8	10	8	8	7
		50.00%	33.30%	41.70%	66.70%	83.30%	66.70%	66.70%	53.80%
	Cohesive	3	2	2	0	0	0	0	2
		25.00%	16.70%	16.70%	0.00%	0.00%	0.00%	0.00%	15.40%
	Mixed	3	6	5	4	2	4	4	4
		25.00%	50.00%	41.70%	33.30%	16.70%	33.30%	33.30%	30.80%
Apical	Adhesive	2	1	5	7	5	4	1	4
		16.70%	8.30%	41.70%	58.30%	41.70%	33.30%	8.30%	30.80%
	Cohesive	2	4	3	2	3	2	7	3
		16.70%	33.30%	25.00%	16.70%	25.00%	16.70%	58.30%	23.10%
	Mixed	8	7	4	3	4	6	4	6
		66.70%	58.30%	25.00%	50.00%	41.70%	33.30%	16.70%	33.30%

**Figure 5:**
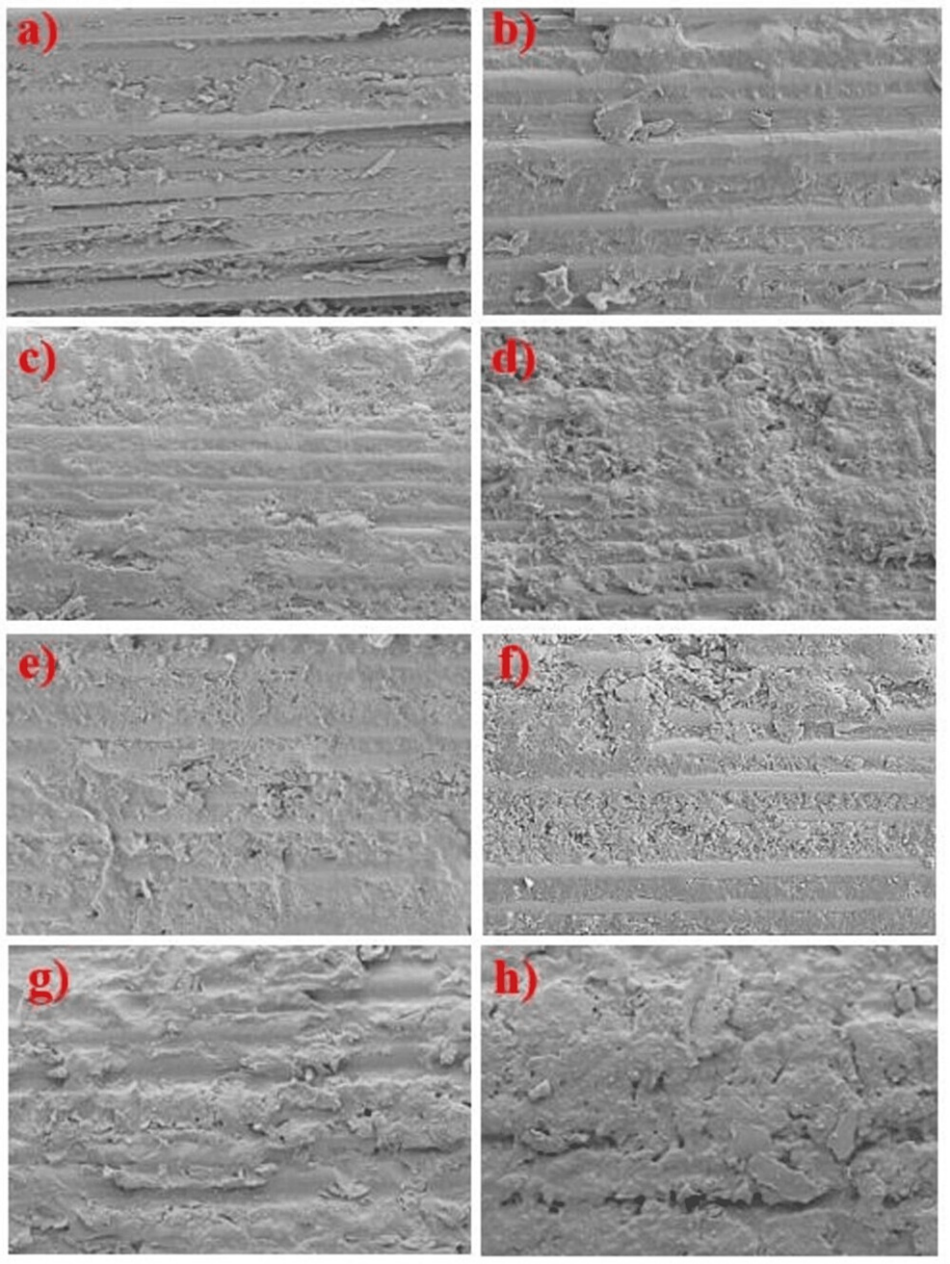
Scanning electron microscope (SEM) images of surface-untreated and treated fiber posts. (a, b) SEM micrographs of surface untreated fiber posts with parallelly oriented fibers. (c, d) SEM micrograph of fiber posts treated with silane showing rough surfaces creating more spaces for micromechanical retention. (e, f) SEM micrograph of fiber posts treated with TiF_4_ and silane shows a layer deposited on the surface of the posts. (g, h) SEM micrograph of fiber posts treated with sandblasting and silane shows a more retentive surface.

SEM analysis results were similar to those of bond strength values. Groups treated with sandblasting and silanization produced more retentive areas, which is in accordance with the highest bond strength values recorded for that group (Figure 5).

**Figure 6:**
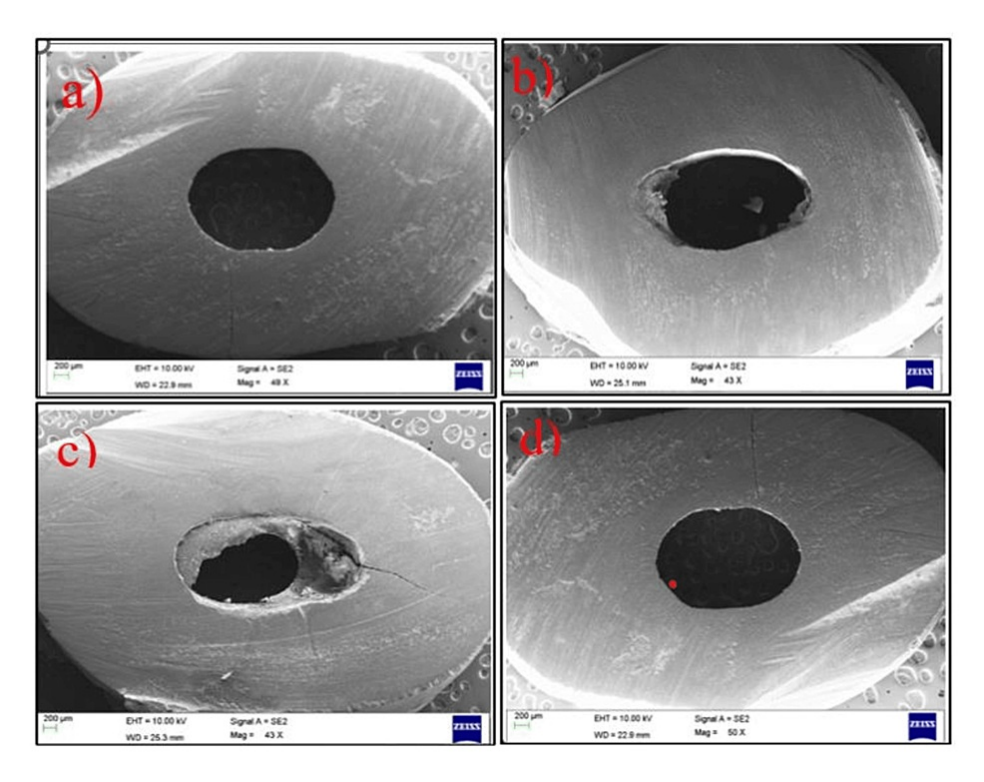
Scanning microscopic confirmation of failure modes. (a) Scanning electron microscope (SEM) micrograph of untreated posts showing adhesive failure between dentin and resin cement. (b) SEM micrograph of fiber posts treated with a silane coupling agent showing mixed failure. (c) SEM micrograph of fiber posts treated with TiF_4_ followed by silane showing cohesive failure within the resin cement. (d) SEM micrograph of fiber posts treated with sandblasting followed by silane showing adhesive failure between the resin cement and dentin.

 Modes of failure seen under stereomicroscope were confirmed by SEM (Figure 6).

## Discussion

In the early 1990s, FRC posts were introduced to substitute for cast post and core to rehabilitate endodontically treated teeth with gross loss of tooth structure [16]. The degree of hydration of root dentin, surface treatment agents of both fiber posts and post space, luting cements, eugenol sealers, configuration factors, and morphologic alterations in solidity and arrangement of dentinal tubules at various levels of the root canal influence the post-root dentin bonding [17].

Adhesive resin cements were used depending on their capacity to bond with root dentin and post, making the luting conservative with a reduction of potential stress [18].

Proper bonding is required at the dentin-cement, post-cement, and post-composite core interfaces for even dissipation of occlusal stresses. The secondary smear layer usually hinders the effective adhesion of the cement to the dentin. Bond failure at any of the interfaces impairs the induction of the monoblock, and the common failure influences debonding at the resin-dentin interface [19]. Hence, our study is focused on improving the bond strength of the fiber posts to the radicular dentin by treating the surface of the posts with various agents.

Various surface treatments have been used to treat the surface of the posts to improve the bond strength, namely, hydrogen peroxide, hydrofluoric acid, methylene chloride, silanization, and airborne particle abrasion [20]. Silanization has been used commonly, but the use of silane to improve the bonding of fiber posts to root dentin remains controversial [15]. In a previous study by Elsaka et al. (2019), 4% TiF_4_ when applied for four minutes exhibited higher bond strength [13]. In our study, 4% TiF_4_ for four minutes was applied followed by silanization, which has not been studied previously.

The push-out test is used in this study because it is closest to clinical conditions [21], more appropriate [22] for evaluating the fiber posts to radicular dentin bonding and it is a true shear test [21]. The impact of anatomic retentive factors might be decreased by sectioning the bonded roots and measuring the bond strength of each specimen by using the push-out method. The comparison of bond strength at different levels of the root can also be made by this method [22].

The present study showed that different surface treatments cause changes on the fiber post surface, the pushout bond strength produced was significantly different between the groups (p < 0.05), and the null hypotheses were rejected. The fiber posts treated with sandblasting followed by silanization produced the highest bond strength in both the glass and quartz groups followed by the silane-treated groups.

In our study, a pre-hydrolyzed silane coupling agent with 3-methacryloxypropyl trimethoxysilane (MPS) was used. The methacryloxyl favors the formation of a chemical bond between the silane-treated quartz fibers and the methacrylate-based matrix of the resin composites, thereby improving the bond strength between them when compared with the untreated surfaces [23]. However, MPS silanes do not form a strong chemical bond between the epoxy resin of the fiber post and the methacrylate resin of the composites. However, they are capable of coupling hydroxyl-covered substrates to the organic matrix of resin adhesives. Such a chemical bond can be achieved only after exposure of the glass/quartz fibers by etching or sandblasting the post surface with alumina particles [24]. Thus, in our study, the fiber posts treated with silane showed higher bond strength than the untreated fiber posts, but the fiber posts treated with sandblasting followed by silanization were the highest in bond strength, which is in accordance with the previous study [25].

Fiber posts (both glass and quartz) treated with 4% TiF_4_ for four minutes followed by silanization produced the lowest bond strength values when compared to fiber posts treated with silane and sandblasting followed by silanization in our study. TiF_4_ acts by removing the superficial layer of the resin matrix and forms a precipitate of titanium on the post surface [13]. The hydrolysis of TiF_4_ occurs on the post surface due to the reaction between titanium and oxygen [26]. For silanization to be effective, the surface of the post generally requires exposed glass fibers, but titanium forms a precipitate over the surface of the exposed glass fibers of the post as examined under SEM in our study, which could explain the lowest bond strength exhibited by the samples treated with 4% TiF_4_ for four minutes followed by silanization [27].

The fiber posts (both glass and quartz) treated with sandblasting followed by silanization showed the highest bond strength of all surface treatments performed in our study, and it was found to be statistically significant. Sandblasting of posts removes the resin matrix between the fibers of the post, making it more retentive. In addition, the combined chemical reaction of silanes, which relies on the formation of Si-O-Si siloxane bonds, improves the surface wettability [27].

Because the post retains and stabilizes the respective core, it is essential to assess the different levels of adhesion of the post to the root dentin [15]. Thus, the evaluation of bond strength at different levels was one of the objectives of our study. In all groups, the bond strength was found to be the highest at the coronal level, and it was found to be statistically significant with the bond strength produced at the apical level. In groups IB, IC, and ID, a statistically significant difference (p < 0.05) was found between the middle and apical root third. The reason for the bond strength being highest at the coronal level may be due to the increased density of dentinal tubules at that level, which increases the penetration of resin into the dentinal tubules. Moreover, dentin hybridization is not uniform in the apical third, and the lateral branches of resin tags were not observed in the apical part of the post-adhesive system [28]. One more reason for the better bond strength at the coronal level may be the ease of application of adhesive agents at that level [15].

In our study, adhesive failures between the resin cement and dentin were found to be predominant in all groups, followed by mixed failures. Bonding to root dentin might be a challenge due to its complex anatomy, handling characteristics of the adhesive systems, and adhesive procedures. The C-factor is critical in a root canal, which increases the polymerization stress of resin cements leading to a decrease in surface energy of the root dentin, resulting in spaces that affect the adhesive interface and compromise the restoration longevity [29].

The surface morphology of the posts examined under SEM after surface treatment was in accordance with the bond strength values. Untreated post surfaces showed evenly distributed glass fibers and unexposed quartz fiber surfaces. Fiber posts treated with silane showed rough surfaces, creating more spaces for micromechanical retention. Fiber posts treated with TiF_4_ followed by silanization showed a layer of precipitate (titanium) [14] formed on the post surfaces. In sandblasting followed by silanization, more retentive areas were seen on the surface of the posts for enhanced micromechanical retention. Similar findings were observed by Soares et al. (2008) [30]. However, it is one limitation of the study that all surface-treated posts were not analyzed under SEM to study their surface morphology.

## Conclusions

In this study, the bond strength of the glass and quartz fiber posts was influenced by different surface treatments. Silane-treated fiber posts with prior sandblasting showed significantly higher push-out bond strength than posts treated with silane (no prior treatments) and posts treated with silane-treated fiber posts with prior TiF_4_ treatment.
